# Pretreatment HALP Score and Survival Outcomes in Patients with Metastatic Renal Cell Carcinoma Receiving First-Line Tyrosine Kinase Inhibitors: A Turkish Oncology Group Kidney Cancer Consortium (TKCC) Study

**DOI:** 10.3390/cancers18132127

**Published:** 2026-06-30

**Authors:** Murat Günaltılı, Hatice Bölek, Esma Kaplan Tüzün, Sura Usta, Safa Can Efil, Aysun Fatma Akkus, Selver Işık, Caner Kapar, Serhat Sekmek, Halil Göksel Güzel, Murad Guliyev, Talha Ozudogru, Sema Sezin Göksu, Özlem Nuray Sever, Çagatay Arslan, Nuri Karadurmuş, Mehmet Ali Nahit Sendur, Deniz Tural, Mustafa Özgüroğlu, Emre Yekedüz, Yüksel Ürün

**Affiliations:** 1 Department of Medical Oncology, Cerrahpasa School of Medicine, Istanbul University-Cerrahpasa, 34098 Istanbul, Turkey; muratgunaltili@gmail.com (M.G.); drmuradguliyev@gmail.com (M.G.); mozgur@iuc.edu.tr (M.Ö.); 2 Department of Medical Oncology, Bilkent City Hospital, 06800 Ankara, Turkey; hati.kocc@gmail.com (H.B.); scefil@hotmail.com (S.C.E.); masendur@yahoo.com.tr (M.A.N.S.); 3 Department of Medical Oncology, Gülhane Training and Research Hospital, University of Health Science, 06010 Ankara, Turkey; esmanurkaplan@hotmail.com (E.K.T.); drnkaradurmus@yahoo.com (N.K.); 4 Department of Medical Oncology, Dr Abdurrahman Yurtaslan Oncology Training and Research Hospital, University of Health Science, 06200 Ankara, Turkey; sura.oztekin@hotmail.com; 5 Department of Medical Oncology, Trakya University School of Medicine, 22030 Edirne, Turkey; aysunfatmadogan@gmail.com; 6 Department of Medical Oncology, Marmara University School of Medicine, 34899 Istanbul, Turkey; selverr83@gmail.com; 7 Department of Medical Oncology, Sakarya Training and Research Hospital, 54100 Sakarya, Turkey; kaparcaner@gmail.com; 8 Department of Medical Oncology, Amasya Training and Research Hospital, 05100 Amasya, Turkey; serhatsekmek@gmail.com; 9 Department of Medical Oncology, Antalya Training and Research Hospital, 06100 Antalya, Turkey; hgguzell@gmail.com; 10 Department of Medical Oncology, Ege University School of Medicine, 35100 Izmir, Turkey; talhaozudogru@hotmail.com; 11 Department of Medical Oncology, Akdeniz University School of Medicine, 07070 Antalya, Turkey; semasezgingoksu@gmail.com; 12 Department of Medical Oncology, Istanbul Sancaktepe Şehit Prof. Dr. İlhan Varank Training and Research Hospital, 34785 Istanbul, Turkey; ozlem.sever@hotmail.com; 13 Medical Point Hospital, Izmir University of Economics, 35575 Izmir, Turkey; arslancagatay@yahoo.com; 14 Department of Medical Oncology, Koc University, 34450 Istanbul, Turkey; deniztural@gmail.com; 15 Department of Medical Oncology, Ankara University School of Medicine, 06230 Ankara, Turkey; emreyekeduz@gmail.com; 16 Ankara University Cancer Institute, 06590 Ankara, Turkey

**Keywords:** metastatic renal cell carcinoma, tyrosine kinase inhibitor, HALP score, overall survival, time to treatment failure

## Abstract

**Background/Objectives:** The hemoglobin, albumin, lymphocyte, and platelet (HALP) score has been investigated as an immunonutritional biomarker in several solid tumors; however, data in metastatic renal cell carcinoma (mRCC) remain limited. This study aimed to evaluate the prognostic significance of the pretreatment HALP score in patients with mRCC receiving first-line tyrosine kinase inhibitor (TKI) therapy. **Methods:** A multicenter retrospective cohort study was conducted using data from the Turkish Oncology Group Kidney Cancer Consortium (TKCC) Database. HALP was calculated as hemoglobin (g/dL) × albumin (g/dL) × lymphocyte count (×10^9^/L)/platelet count (×10^9^/L). Patients were categorized into HALP-low and HALP-high groups according to the cohort median HALP value. **Results:** A total of 1001 patients were enrolled. The median time to treatment failure (TTF) was significantly shorter in the HALP-low group than in the HALP-high group (7.43 months [95% CI 6.4–8.5] vs. 14.26 months [95% CI 12.1–16.4], *p* < 0.001). The median overall survival (OS) was also significantly shorter in the HALP-low group (30.19 months [95% CI 25.2–35.1] vs. 46.06 months [95% CI 38.2–53.9], *p* < 0.001). In multivariable analysis, a high HALP score remained independently associated with longer TTF (HR 0.73, 95% CI 0.62–0.86; *p* < 0.001) and OS (HR 0.69, 95% CI 0.57–0.85; *p* < 0.001). **Conclusions:** The pretreatment HALP score was independently associated with TTF and OS in patients with mRCC treated with first-line TKI therapy. These findings support the prognostic relevance of HALP in this treatment setting and warrant further evaluation of its potential role alongside established clinical risk models.

## Simple Summary

Prognostic assessment in metastatic renal cell carcinoma may be improved by incorporating simple markers that reflect both tumor-related systemic inflammation and the general condition of the patient. The hemoglobin, albumin, lymphocyte, and platelet (HALP) score combines routinely measured parameters related to anemia, nutritional status, immune response, and inflammation. In this large, multicenter, real-world study, we evaluated the prognostic value of pretreatment HALP in patients with metastatic renal cell carcinoma receiving first-line tyrosine kinase inhibitor (TKI) therapy. The HALP-low group had a significantly shorter time to treatment failure and overall survival than the HALP-high group. These findings suggest that HALP may be a useful prognostic marker in patients receiving first-line TKI therapy and support its further evaluation in future studies.

## 1. Introduction

Renal cell carcinoma (RCC) is the predominant form of kidney malignancy in adults and represents a biologically and clinically heterogeneous group of tumors [1]. Approximately 20–25% of patients present with metastatic disease at diagnosis, while a considerable proportion of those initially diagnosed with localized disease and treated with curative intent subsequently develop recurrence during follow-up [2,3]. Significant advances have been made in the treatment of metastatic renal cell carcinoma (mRCC) using tyrosine kinase inhibitors (TKIs) and immune checkpoint inhibitors. Although immune checkpoint inhibitor-based combinations are recommended in the first-line setting, TKI monotherapy remains relevant in selected patients and in real-world practice [4,5,6]. However, even among patients with similar clinical characteristics and in the same risk group, treatment response and survival outcomes may differ markedly. This variability suggests that currently available prognostic tools may not fully capture the biological heterogeneity of the disease [7,8]. Although the International Metastatic RCC Database Consortium (IMDC) model remains the most commonly used risk stratification system in routine practice, there is a continuing need for additional easily accessible biomarkers that may better reflect prognosis and clinical outcomes [9,10,11].

In mRCC, tumor biology is associated not only with the extent of the disease but also with systemic processes that reflect host response [12]. Systemic inflammation is an important mechanism that supports the proliferation of tumor cells, angiogenesis, and escape from the immune system [13,14]. In immunologically active tumors, such as RCC, inflammatory signaling and cytokine release within the tumor microenvironment have been shown to influence survival outcomes [15,16,17]. Therefore, various inflammatory markers, including the neutrophil-to-lymphocyte ratio, platelet-to-lymphocyte ratio, and systemic immune–inflammation index, have been reported to correlate with survival and prognosis [18,19]. In addition, some routine laboratory parameters underlying these markers have been shown to have prognostic significance. Low hemoglobin levels and thrombocytosis, both evaluated in the IMDC risk classification, are considered adverse prognostic features [20]. The effects of inflammation are not limited to immune cells alone but are also closely related to the patient’s nutritional and metabolic status. For example, albumin, a negative acute-phase reactant synthesized by the liver, is a useful indicator of malnutrition and systemic effects of severe inflammation. Similarly, lymphocyte count has been evaluated as an indirect indicator of the antitumor immune response, and low lymphocyte levels have been shown to be associated with a poor prognosis [16,21,22]. Taken together, hemoglobin, albumin, lymphocyte, and platelet levels are readily available and cost-effective components that reflect the interplay among inflammation, nutrition, and immunity in patients with cancer.

The hemoglobin–albumin–lymphocyte–platelet (HALP) score, derived from the combined assessment of these four variables, has been investigated across several solid tumors in recent years, and low HALP values have been associated with shorter survival and worse prognosis [23,24,25]. In renal cell carcinoma specifically, studies examining the prognostic role of HALP have also been reported, and low HALP scores have been linked to unfavorable clinical outcomes in both metastatic disease and post-nephrectomy cohorts [26,27,28]. Nevertheless, current data in this field remain limited, and there is significant heterogeneity among studies in terms of patient populations, treatment options, and the cut-off values used to define HALP categories. Therefore, in the present study, we aimed to investigate the prognostic impact of the pretreatment HALP score on time to treatment failure (TTF) and overall survival (OS) in a large, multicenter cohort of patients with mRCC who received first-line tyrosine kinase inhibitor therapy.

## 2. Materials and Methods

### 2.1. Study Design and Patient Population

This multicenter retrospective cohort study was conducted using data from the Turkish Oncology Group Kidney Cancer Consortium (TKCC) database. The TKCC database is a multi-institutional retrospective registry that includes clinical, pathological, treatment-related, and survival data from participating oncology centers in Turkey. Data are periodically reviewed for completeness and consistency, and queries are resolved with the contributing centers when needed. Adult patients (≥18 years) with mRCC who initiated first-line TKI therapy between June 2007 and July 2024 were included. To be included, all laboratory variables required for the calculation of the HALP score had to be available within 15 days before the initiation of first-line TKI treatment. For patients with multiple available measurements, the values closest to treatment initiation were used.

Demographic, clinical, pathological, and treatment-related data were obtained from the TKCC database. The collected variables included age at metastatic diagnosis, sex, comorbidity status, Eastern Cooperative Oncology Group (ECOG) performance status, histologic subtype, sarcomatoid differentiation, International Metastatic RCC Database Consortium (IMDC) risk group, and metastatic sites.

### 2.2. HALP Assessment and Outcome Definitions

The HALP score was calculated as hemoglobin (g/dL) × albumin (g/dL) × lymphocyte count (×10^9^/L)/platelet count (×10^9^/L). Patients were categorized into HALP-low and HALP-high groups according to the cohort median HALP value; values less than or equal to the median were classified as HALP-low, and values above the median as HALP-high.

The study endpoints were TTF and OS. TTF was defined as the time from the initiation of first-line tyrosine kinase inhibitor therapy to disease progression, death, or treatment discontinuation for any reason, whichever occurred first. OS was defined as the time from treatment initiation to death from any cause.

### 2.3. Statistical Analysis

Statistical analyses were performed using IBM SPSS Statistics version 24.0 (IBM Corp., Armonk, NY, USA). Continuous variables are summarized as medians with interquartile ranges (IQRs), whereas categorical variables are presented as counts and percentages. Group comparisons were performed using the chi-square test for categorical variables and Student’s *t*-test or Mann–Whitney U test for continuous variables, as appropriate. Survival outcomes were estimated using the Kaplan–Meier method and compared using the log-rank test. Variables showing an association at a threshold of *p* < 0.10 in the univariate analyses were entered into the multivariable Cox proportional hazards model, and hazard ratios (HRs) with 95% confidence intervals (CIs) are reported. Because the IMDC model includes laboratory variables that overlap with the components of the HALP score, the IMDC risk category was not included in the multivariable analysis. To assess the robustness of the findings after accounting for the established IMDC classification, additional sensitivity analyses were performed by including the IMDC risk group in the multivariable Cox models for TTF and OS. Model discrimination was assessed using the concordance index (C-index). C-index values with 95% confidence intervals were calculated for the IMDC model alone and for the model combining IMDC with HALP. These analyses were performed using Python (https://www.python.org/, accessed on 25 June 2026). All tests were two-sided, and a *p*-value below 0.05 was considered statistically significant.

### 2.4. Ethical Considerations

This retrospective study was conducted in accordance with the Declaration of Helsinki and was approved by the Ankara University Medical Faculty Ethics Committee (Approval Number: I09-701-24, Date: 11 September 2024). The requirement for informed consent for participation was waived by the Ethics Committee because of the retrospective design of the study.

## 3. Results

### 3.1. Baseline Patient Characteristics

Among the 1622 patients with metastatic RCC who received systemic treatment during the study period, 1134 received first-line TKI therapy. Of these, 127 were excluded because at least one laboratory parameter required for calculating the HALP score was unavailable, and six were excluded because survival data were incomplete. The final study population comprised 1001 patients. Patient selection is summarized in Supplementary Figure S1. The median age of the cohort was 61.5 years (IQR: 15.1), and 71.7% of patients were male. A total of 803 patients (80.2%) had an ECOG performance status of 0–1, and clear-cell histology was the predominant subtype, accounting for 780 cases (77.9%). Sunitinib was the most commonly used first-line treatment (55.6%), followed by pazopanib (36.9%) and cabozantinib (7.5%) (Table 1).

The median HALP score was 0.274, and patients were categorized into HALP-low (*n* = 501) and HALP-high (*n* = 500) groups according to this value. Compared with the HALP-high group, patients in the HALP-low group were older (median age, 62.5 [IQR, 15.9] vs. 60.4 [IQR, 13.5] years, *p* = 0.001), more likely to have an ECOG performance status of ≥2 (24.2% vs. 11.4%, *p* < 0.001), and more frequently classified as having a poor risk according to the IMDC model (38.7% vs. 7.6%, *p* < 0.001). The prevalence of specific metastatic sites also varied; the HALP-low group had higher rates of bone metastasis (47.7% vs. 31.2%, *p* < 0.001) and liver involvement (23.4% vs. 13.8%, *p* < 0.001) than the HALP-high group. All baseline patient characteristics and group comparisons are presented in Table 1.

### 3.2. Survival Outcomes

**Figure 1:**
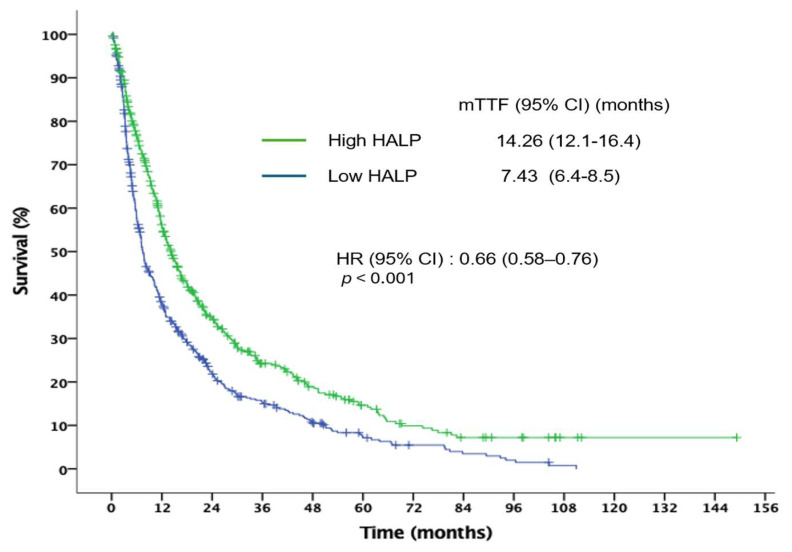
Kaplan–Meier curve for time to treatment failure according to the HALP group in patients with metastatic renal cell carcinoma treated with first-line tyrosine kinase inhibitors. Abbreviations: CI, confidence interval; HALP, hemoglobin–albumin–lymphocyte–platelet; TTF, time to treatment failure.

For the entire cohort, the median time to treatment failure (TTF) was 10.91 months [95% CI, 9.7–12.1]. Patients with low HALP scores had a significantly shorter median TTF than those with high HALP scores (7.43 months [95% CI, 6.4–8.5] vs. 14.26 months [95% CI, 12.1–16.4], *p* < 0.001) (Figure 1).

In the univariate analysis of TTF, several variables were associated with shorter TTF, including age ≥65 years, poor performance status (ECOG ≥ 2), presence of sarcomatoid features, poor IMDC risk classification, and metastatic involvement of the liver, lung, bone, and lymph nodes/soft tissue. In addition, a high HALP score was significantly associated with a longer TTF (HR 0.66, 95% CI 0.58–0.76; *p* < 0.001). In multivariate analysis, HALP remained an independent prognostic factor for TTF (HR 0.734, 95% CI 0.622–0.865; *p* < 0.001) after adjustment for age, ECOG performance status, sarcomatoid features, and the presence of lung, liver, lymph node/soft tissue, and bone metastases (Table 2).

**Figure 2:**
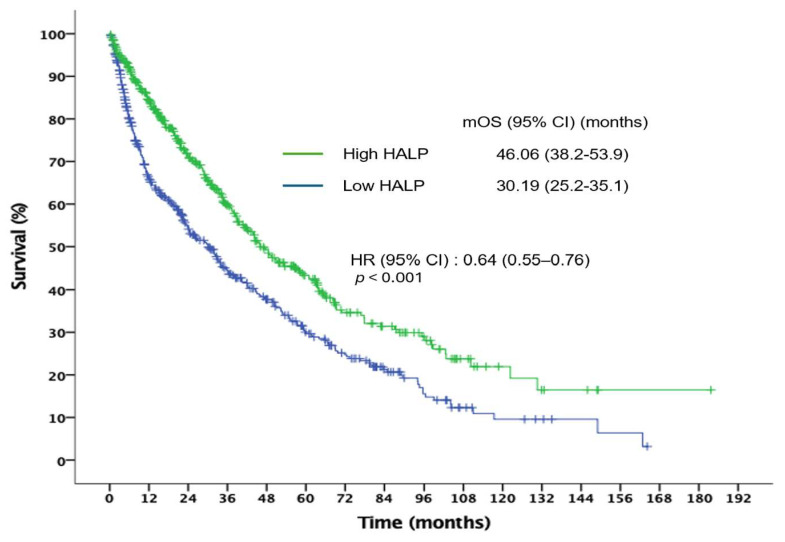
Kaplan–Meier curve for overall survival according to the HALP group in patients with metastatic renal cell carcinoma treated with first-line tyrosine kinase inhibitors. Abbreviations: CI, confidence interval; HALP, hemoglobin–albumin–lymphocyte–platelet; OS, overall survival.

The median OS for the entire cohort was 37.89 months [95% CI, 32.7–43.1]. Patients with low HALP scores had a significantly shorter median OS than those with high HALP scores (30.19 months [95% CI, 25.2–35.1] vs. 46.06 months [95% CI, 38.2–53.9], *p* < 0.001) (Figure 2).

In the univariate analysis of OS, age ≥65 years, ECOG performance status of ≥2, poor IMDC risk classification, and the presence of liver, bone, or lymph node/soft tissue metastases were associated with a shorter OS. In addition, a high HALP score was significantly associated with longer OS (HR 0.64, 95% CI 0.55–0.76; *p* < 0.001). Multivariate analysis showed that HALP was an independent prognostic factor for OS (HR 0.69, 95% CI 0.57–0.85; *p* < 0.001) after adjustment for age, ECOG performance status, presence of sarcomatoid features, liver metastasis, lymph node or soft tissue metastasis, bone metastasis, and first-line treatment (Table 3).

Regarding individual first-line TKI agents, no significant differences in TTF were observed between pazopanib or cabozantinib and sunitinib. For OS, cabozantinib was associated with shorter survival than sunitinib in univariate analysis, but this association was not retained in the multivariate model. Pazopanib was not clearly associated with a difference in OS compared with sunitinib.

In sensitivity analyses including the IMDC risk group, a high HALP score remained independently associated with longer TTF (HR 0.80, 95% CI 0.66–0.97; *p* = 0.021). For OS, the association was attenuated and did not reach statistical significance (HR 0.82, 95% CI 0.66–1.03; *p* = 0.097) (Supplementary Table S1). In additional discrimination analyses, the C-index for OS was 0.601 (95% CI, 0.576–0.624) for the IMDC model alone and increased to 0.616 (95% CI, 0.589–0.641) when HALP was added to the model. For TTF, the C-index increased from 0.590 (95% CI, 0.570–0.610) for the IMDC model alone to 0.604 (95% CI, 0.581–0.625) for the model combining IMDC with HALP.

## 4. Discussion

The present study represents one of the largest multicenter analyses evaluating the prognostic significance of the HALP score in patients with mRCC receiving first-line TKI therapy. In this population, a low HALP score was independently associated with shorter TTF and OS. The study period reflects real-world clinical practice in Turkey, where immune checkpoint inhibitor-based combinations were not routinely reimbursed as first-line treatment for mRCC during the inclusion period. Although immune checkpoint inhibitor-based combinations are now the preferred first-line approach, TKI monotherapy remains relevant for selected patients who are not candidates for immunotherapy, and VEGFR-TKIs continue to play an important role in subsequent treatment lines. These findings suggest that the HALP score may serve as a practical and readily applicable prognostic indicator in patients with mRCC treated with first-line TKIs.

In recent years, the HALP score has emerged as a biomarker reflecting the immunonutritional status of patients across various solid malignancies. In a large pooled analysis including more than 17,049 patients, Li et al. showed that a higher HALP score was associated with better survival outcomes in patients with different solid tumors [29]. Likewise, Farag et al. reported that the HALP score may have prognostic relevance across several cancer types [30].

However, the evidence specific to RCC is limited. Existing RCC studies have largely focused on cohorts undergoing curative surgery or relatively small metastatic populations. Peng et al. evaluated the prognostic impact of the HALP score in 1360 patients with RCC treated with nephrectomy and demonstrated that a low HALP score was associated with shorter cancer-specific survival [26]. Among studies conducted on mRCC, Ekinci et al. reported an association between a low HALP score and poor prognosis in a metastatic cohort of 123 patients [27]. However, the treatment context was not described in sufficient detail to determine whether the cohort represented a homogeneous systemic therapy setting. Köşeci et al. evaluated the HALP score as part of a combined prognostic model in a cohort of 147 patients receiving targeted therapy, although the distribution of individual TKI agents and the exact treatment line were not clearly reported [31]. Studies in nivolumab-treated mRCC populations have also suggested an association between low HALP scores and shorter survival outcomes [28,32]. Against this background, our study adds to the existing literature by demonstrating the prognostic significance of the HALP score in a large, relatively homogeneous, multicenter cohort of patients with metastatic RCC treated with first-line TKI monotherapy.

Because no validated HALP cut-off has been established for mRCC, we used the cohort median to provide an outcome-independent threshold and balanced group sizes. Although ROC-based methods can identify endpoint-specific cut-offs, such thresholds may be sensitive to the characteristics of the study population when derived and evaluated within the same cohort. Notably, our median HALP value of 0.274 was very close to the ROC-derived cut-off of 0.277 reported by Ekinci et al. [27]. After accounting for the different units used for hemoglobin and albumin, the median value of 27.53 reported by Tomčová et al. corresponds to approximately 0.2753 on the scale used in the present study [28]. This consistency supports the plausibility of the threshold used in our analysis, although prospective validation is required before it can be considered a clinically established cut-off.

The strong prognostic impact of the HALP score in patients with mRCC may be explained by its ability to integrate hemoglobin, albumin, lymphocytes, and platelets, which together capture systemic inflammation, as well as the patient’s nutritional and immunological status. Hemoglobin primarily reflects anemia and tissue oxygen-carrying capacity, and anemia in RCC may be associated with more aggressive tumor biology through hypoxia-driven pathways, including HIF-related signaling [33,34]. Albumin reflects nutritional reserve and the systemic catabolic effects of inflammation, and low albumin levels have been associated with poor prognosis in RCC [35,36]. Lymphocyte count provides an indirect measure of antitumor immune competence. In contrast, elevated platelet counts may contribute to tumor-associated inflammation, angiogenesis, and metastatic progression; both lymphopenia and thrombocytosis have been associated with adverse outcomes in RCC [37,38,39]. Our findings suggest that HALP may serve as a practical marker for the combined impact of systemic inflammation, poor nutritional status, and weakened antitumor immune response in patients with mRCC.

One of the more notable findings of our study was that patients with low HALP scores tended to have less favorable baseline characteristics, including older age, poorer performance status, and less favorable patterns of metastatic involvement. In particular, the higher frequency of liver and bone metastases in the HALP-low group suggests that this score may reflect not only inflammation and nutritional status but also a clinical background associated with more aggressive disease biology. Data on the relationship between HALP score and age are highly heterogeneous. Several studies have associated older age with higher HALP scores, or conversely, younger age with lower HALP levels [40,41,42]. In contrast, Peng et al. found that lower HALP levels were associated with older age, consistent with our results [26]. Regarding performance status, the association between low HALP and poorer ECOG performance status observed in our study is also consistent with the findings reported by Vlatka et al. in patients with lymphoma [43]. In smaller metastatic RCC cohorts, no clear association was demonstrated between metastatic sites and HALP score, whereas patients with low HALP scores in our cohort more frequently had bone and liver metastases [28,32]. They were also more frequently classified as having poor IMDC risk. These findings suggest that HALP may reflect not only immunonutritional status but also broader clinical frailty and disease burden.

In the sensitivity analysis including the IMDC risk group, HALP remained independently associated with TTF, whereas its association with OS was attenuated. In the OS model, neither HALP nor IMDC retained statistical significance. The shared inclusion of hemoglobin and platelet count in both assessments may have reduced their apparent independent contributions when evaluated within the same model. In additional discrimination analyses, the addition of HALP to the IMDC classification resulted in modest numerical increases in the C-index for both TTF and OS. These findings suggest that HALP may provide complementary prognostic information beyond the IMDC classification, although the observed incremental improvement requires validation in independent cohorts.

Our study had several important strengths. To our knowledge, this study represents one of the largest multicenter cohorts evaluating the prognostic value of the HALP score in patients with mRCC treated with first-line TKI therapy. The multicenter design, large sample size, and consistent first-line TKI treatment context strengthened the statistical robustness and real-world relevance of the findings while reducing treatment-related heterogeneity. These findings may also provide a reference for future studies evaluating HALP in patients receiving contemporary immune checkpoint inhibitor-based combinations. In addition, the HALP score is a low-cost and easily applicable index derived from routinely available laboratory parameters, which enhances its potential utility in daily oncology practice. Finally, one of the distinctive contributions of our study is that the HALP score was associated not only with overall survival but also with time to treatment failure.

Nevertheless, several limitations should be considered when interpreting the findings. First, the retrospective design of the study inherently introduces the possibility of selection bias. In addition, the HALP score was assessed only at a single time point before treatment initiation, and dynamic changes during therapy and their potential prognostic relevance were not evaluated. Another important limitation is the absence of a universally accepted cut-off value for the HALP score. The variability in cut-off values across studies, depending on the population and methodology used, makes direct comparisons between studies difficult. Finally, the exclusive inclusion of patients receiving first-line TKI monotherapy limits the applicability of the findings to the contemporary mRCC population. Therefore, the results should not be directly extrapolated to patients receiving immune checkpoint inhibitor-based combinations or TKIs after prior immunotherapy, and the prognostic relevance of HALP should be validated separately in these treatment settings.

## 5. Conclusions

The pretreatment HALP score was independently associated with TTF and OS in patients with mRCC treated with first-line TKIs. Our findings support the potential prognostic value of the HALP score in this population. Prospective studies are warranted to confirm these results, establish an optimal cut-off for clinical use, and determine whether the HALP score retains its prognostic utility in the era of immune checkpoint inhibitor-based combination treatment.

## Figures and Tables

**Table 1:** Baseline demographic, clinical, and treatment characteristics according to the HALP group.

	All Patients N = 1001 (%)	HALP-Low Group N = 501 (%)	HALP-High Group N = 500 (%)	*p*-Value
Age (years), median (IQR)	61.5 (15.1)	62.5 (15.9)	60.4 (13.5)	0.001
Age category				
<65 years	644 (64.3)	305 (60.9)	339 (67.8)	0.022
≥65 years	357 (35.7)	196 (39.1)	161 (32.2)	
Gender				
Female	283 (28.3)	161 (32.1)	122 (24.4)	0.008
Male	718 (71.7)	340 (67.9)	378 (75.6)	
ECOG PS				
0–1	803 (80.2)	370 (73.9)	433 (86.6)	
≥2	178 (17.8)	121 (24.2)	57 (11.4)	<0.001
Unknown	20 (2.0)	10 (2.0)	10 (2.0)	
Histology				
Clear cell	780 (77.9)	385 (76.8)	395 (79.0)	
Non-clear cell	170 (17.0)	85 (17.0)	85 (17.0)	0.880
Unknown	51 (5.1)	31 (6.2)	20 (4.0)	
Sarcomatoid differentiation				
Absent	655 (65.4)	317 (63.3)	338 (67.6)	
Present	107 (10.7)	58 (11.6)	49 (9.8)	0.265
Unknown	239 (23.9)	126 (25.1)	113 (22.6)	
IMDC risk group				
Favorable	146 (14.6)	20 (4.0)	126 (25.2)	
Intermediate	507 (50.6)	242 (48.3)	265 (53)	<0.001
Poor	232 (23.2)	194 (38.7)	38 (7.6)	
Unknown	116 (11.6)	45 (9.0)	71 (14.2)	
Metastatic sites				
Lung metastasis	671 (67)	344 (68.7)	327 (65.4)	0.272
Liver metastasis	186 (18.6)	117 (23.4)	69 (13.8)	<0.001
Bone metastasis	395 (39.5)	239 (47.7)	156 (31.2)	<0.001
Soft tissue/lymph node metastasis	581 (58.0)	308 (61.5)	273 (54.6)	0.027
First-line therapy				
Sunitinib	557 (55.6)	249 (49.7)	308 (61.6)	
Pazopanib	369 (36.9)	209 (41.7)	160 (32)	<0.001
Cabozantinib	75 (7.5)	43 (8.6)	32 (6.4)	

ECOG PS, Eastern Cooperative Oncology Group performance status; HALP, hemoglobin, albumin, lymphocyte, and platelet; IMDC, International Metastatic RCC Database Consortium; IQR, interquartile range.

**Table 2:** Univariate and multivariate Cox regression analyses for predictors of time to treatment failure.

Variable		Univariate Analyses HR (95% CI)	*p*-Value	Multivariate Analyses HR (95% CI)	*p*-Value
Age	<65	1		1	
	≥65	1.34 (1.16–1.55)	<0.001	1.20 (1.01–1.42)	0.039
Gender	Female	1			
	Male	1.08 (0.92–1.26)	0.354		
ECOG PS	0–1	1		1	
	≥2	1.51 (1.27–1.80)	< 0.001	1.27 (1.03–1.58)	0.026
Histologic subtype	Clear cell	1			
	Non-clear cell	1.14 (0.95–1.37)	0.166		
Sarcomatoid features	No	1		1	
	Yes	1.43 (1.15–1.78)	0.001	1.39 (1.11–1.73)	0.004
IMDC risk group	Favorable	1			
	Poor	1.33 (1.23–1.43)	<0.001		
Lung metastasis	No	1		1	
	Yes	1.22 (1.05–1.41)	0.010	1.26 (1.06–1.49)	0.008
Liver metastasis	No	1		1	
	Yes	1.57 (1.32–1.86)	<0.001	1.56 (1.28–1.90)	<0.001
Soft tissue/lymph node metastasis	No	1		1	
	Yes	1.27 (1.10–1.46)	0.001	1.22 (1.03–1.44)	0.018
Bone metastasis	No	1		1	
	Yes	1.28 (1.11–1.47)	0.001	1.26 (1.07–1.49)	0.007
First-line treatment	Sunitinib	1			
	Pazopanib	0.94 (0.82–1.09)	0.437		
	Cabozantinib	0.90 (0.64–1.25)	0.521		
HALP score	Low	1		1	
	High	0.66 (0.58–0.76)	<0.001	0.73 (0.62–0.86)	<0.001

CI, confidence interval; ECOG PS, Eastern Cooperative Oncology Group performance status; HALP, hemoglobin, albumin, lymphocyte, and platelet; HR, hazard ratio; IMDC, International Metastatic RCC Database Consortium.

**Table 3:** Univariate and multivariate Cox regression analyses for predictors of overall survival.

Variable		Univariate Analyses HR (95% CI)	*p*-Value	Multivariate Analyses HR (95% CI)	*p*-Value
Age	<65	1		1	
	≥65	1.26 (1.06–1.49)	0.008	1.40 (1.14–1.71)	0.001
Gender	Female	1			
	Male	0.99 (0.83–1.20)	0.978		
ECOG PS	0–1	1		1	
	≥2	1.39 (1.14–1.69)	0.001	1.42 (1.11–1.80)	0.004
Histologic subtype	Clear cell	1			
	Non-clear cell	1.15 (0.92–1.44)	0.227		
Sarcomatoid features	No	1		1	
	Yes	1.29 (0.99–1.66)	0.055	1.28 (0.98–1.66)	0.061
IMDC risk group	Favorable	1			
	Poor	2.29 (1.70–3.07)	<0.001		
Lung metastasis	No	1			
	Yes	1.11 (0.93–1.33)	0.243		
Liver metastasis	No	1		1	
	Yes	1.77 (1.46–2.15)	<0.001	1.92 (1.53–2.42)	<0.001
Soft tissue/lymph node metastasis	No	1		1	
	Yes	1.25 (1.06–1.48)	0.008	1.24 (1.02–1.51)	0.034
Bone metastasis	No	1		1	
	Yes	1.37 (1.16–1.62)	<0.001	1.27 (1.03–1.55)	0.022
First-line treatment	Sunitinib	1		1	
	Pazopanib	1.04 (0.87–1.24)	0.665	0.81 (0.66–1.00)	0.050
	Cabozantinib	1.59 (1.07–2.38)	0.022	1.38 (0.86–2.24)	0.181
HALP score	Low	1		1	
	High	0.64 (0.55–0.76)	<0.001	0.69 (0.57–0.85)	<0.001

CI, confidence interval; ECOG PS, Eastern Cooperative Oncology Group performance status; HALP, hemoglobin, albumin, lymphocyte, and platelet; HR, hazard ratio; IMDC, International Metastatic RCC Database Consortium.

## Data Availability

The datasets generated and/or analyzed during the current study are available from the corresponding author upon reasonable request.

## Supplementary Materials

The following supporting information can be downloaded at: https://www.mdpi.com/article/10.3390/cancers18132127/s1, Supplementary Figure S1. Flow diagram of patient selection. Abbreviations: HALP, hemoglobin, albumin, lymphocyte, and platelet score; mRCC, metastatic renal cell carcinoma; TKI, tyrosine kinase inhibitor. Supplementary Table S1. Sensitivity multivariate Cox regression analyses including the IMDC risk group for time to treatment failure (TTF) and overall survival (OS).

## Abbreviations

The following abbreviations are used in this manuscript:

CI	Confidence interval
ECOG PS	Eastern Cooperative Oncology Group performance status
HALP	Hemoglobin, albumin, lymphocyte, and platelet
HR	Hazard ratio
IMDC	International Metastatic Renal Cell Carcinoma Database Consortium
IQR	Interquartile range
mRCC	Metastatic renal cell carcinoma
OS	Overall survival
RCC	Renal cell carcinoma
TKCC	Turkish Oncology Group Kidney Cancer Consortium
TKI	Tyrosine kinase inhibitor
TTF	Time to treatment failure
