# Supplementary material for: Pretreatment HALP Score and Survival Outcomes in Patients with Metastatic Renal Cell Carcinoma Receiving First-Line Tyrosine Kinase Inhibitors: A Turkish Oncology Group Kidney Cancer Consortium (TKCC) Study
Source: Cancers (Basel). 2026 Jun 30;18(13):2127. doi: 10.3390/cancers18132127 (PMC13359624; doi:10.3390/cancers18132127)
Supplement: Supplementary file 1 [file cancers-18-02127-s001.zip › Supplementary Table S1.pdf]

Supplementary Table S1. Sensitivity multivariate Cox regression analyses including the IMDC risk group for time to treatment failure (TTF) and overall survival (OS)

**Variable**		**Multivariate Analyses for TTF HR (95% CI)**	***p*-value**	**Multivariate Analyses for OS HR (95% CI)**	***p*-value**
Age	<65	1		1	
	≥ 65	1.22 (1.02–1.46)	0.029	1.42 (1.14–1.76)	0.001
ECOG PS	0-1	1		1	
	≥2	1.15 (0.90–1.46)	0.259	1.32 (0.99–1.74)	0.052
Sarcomatoid features	No	1		1	
	Yes	1.28 (1.01–1.63)	0.040	1.22 (0.92–1.61)	0.170
IMDC risk group	Favorable	1		1	
	Poor	1.57 (1.16–2.14)	0.004	1.42 (0.87–2.33)	0.163
Lung metastasis	No	1			
	Yes	1.23 (1.03–1.47)	0.026		
Liver metastasis	No	1		1	
	Yes	1.64 (1.33–2.02)	< 0.001	2.03 (1.59–2.58)	< 0.001
Soft tissue / lymph node metastasis	No	1		1	
	Yes	1.25 (1.05–1.49)	0.013	1.23 (0.99–1.52)	0.057
Bone metastasis	No	1		1	
	Yes	1.25 (1.05–1.49)	0.014	1.22 (0.98–1.51)	0.064
First-line treatment	Sunitinib			1	
	Pazopanib			0.79 (0.64–0.99)	0.042
	Cabozantinib			1.42 (0.87–2.33)	0.163
HALP score	Low	1		1	
	High	0.80 (0.66–0.97)	0.021	0.82 (0.66–1.03)	0.097

CI, confidence interval; ECOG PS, Eastern Cooperative Oncology Group performance status; HALP, hemoglobin, albumin, lymphocyte, and platelet; HR, hazard ratio; IMDC, International Metastatic RCC Database Consortium; OS, overall survival; TTF, time to treatment failure.
