# Supplementary material for: Pretreatment HALP Score and Survival Outcomes in Patients with Metastatic Renal Cell Carcinoma Receiving First-Line Tyrosine Kinase Inhibitors: A Turkish Oncology Group Kidney Cancer Consortium (TKCC) Study
Source: Cancers (Basel). 2026 Jun 30;18(13):2127. doi: 10.3390/cancers18132127 (PMC13359624; doi:10.3390/cancers18132127)
Supplement: Supplementary file 1 [file cancers-18-02127-s001.zip › Supplementary Figure S1.pdf]

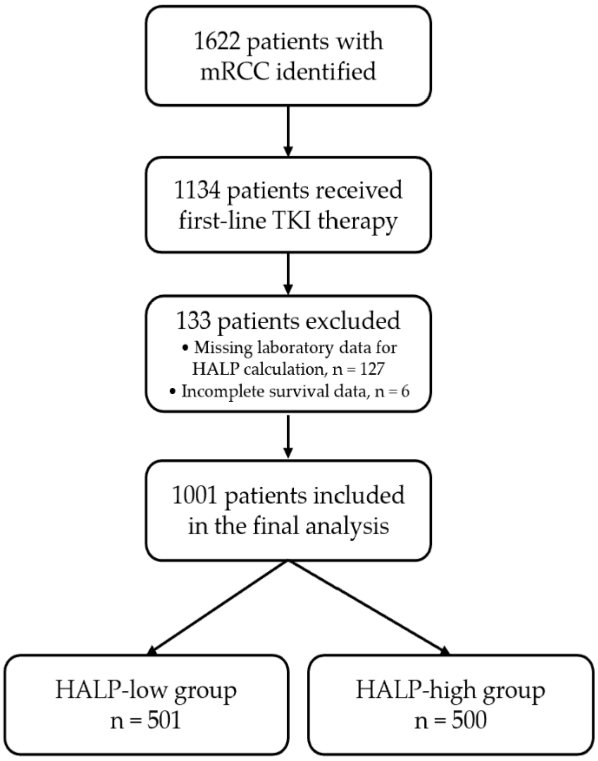

Supplementary Figure S1. Flow diagram of patient selection. Abbreviations: HALP, hemoglobin, albumin, lymphocyte, and platelet score; mRCC, metastatic renal cell carcinoma; TKI, tyrosine kinase inhibitor.
